# Titanium Dioxide Nanoparticles Trigger Loss of Function and Perturbation of Mitochondrial Dynamics in Primary Hepatocytes

**DOI:** 10.1371/journal.pone.0134541

**Published:** 2015-08-06

**Authors:** Vaishaali Natarajan, Christina L. Wilson, Stephen L. Hayward, Srivatsan Kidambi

**Affiliations:** 1 Department of Chemical and Biomolecular Engineering, University of Nebraska-Lincoln, NE, 68588, United States of America; 2 Nebraska Center for Materials and Nanoscience, University of Nebraska-Lincoln, NE, 68588, United States of America; 3 Mary and Dick Holland Regenerative Medicine Program, University of Nebraska Medical Center, NE, 68198, United States of America; Universidad Pablo de Olavide, Centro Andaluz de Biología del Desarrollo-CSIC , SPAIN

## Abstract

Titanium dioxide (TiO_2_) nanoparticles are one of the most highly manufactured and employed nanomaterials in the world with applications in copious industrial and consumer products. The liver is a major accumulation site for many nanoparticles, including TiO_2_, directly through intentional exposure or indirectly through unintentional ingestion via water, food or animals and increased environmental contamination. Growing concerns over the current usage of TiO_2_ coupled with the lack of mechanistic understanding of its potential health risk is the motivation for this study. Here we determined the toxic effect of three different TiO_2_ nanoparticles (commercially available rutile, anatase and P25) on primary rat hepatocytes. Specifically, we evaluated events related to hepatocyte functions and mitochondrial dynamics: (1) urea and albumin synthesis using colorimetric and ELISA assays, respectively; (2) redox signaling mechanisms by measuring reactive oxygen species (ROS) production, manganese superoxide dismutase (MnSOD) activity and mitochondrial membrane potential (MMP); (3) OPA1 and Mfn-1 expression that mediates the mitochondrial dynamics by PCR; and (4) mitochondrial morphology by MitoTracker Green FM staining. All three TiO_2_ nanoparticles induced a significant loss (p < 0.05) in hepatocyte functions even at concentrations as low as 50 ppm with commercially used P25 causing maximum damage. TiO_2_ nanoparticles induced a strong oxidative stress in primary hepatocytes. TiO_2_ nanoparticles exposure also resulted in morphological changes in mitochondria and substantial loss in the fusion process, thus impairing the mitochondrial dynamics. Although this study demonstrated that TiO_2_ nanoparticles exposure resulted in substantial damage to primary hepatocytes, more *in vitro* and *in vivo* studies are required to determine the complete toxicological mechanism in primary hepatocytes and subsequently liver function.

## Introduction

Engineered nanoparticles form a major fraction of man-made nanomaterials currently escalating in both development and commercial implementation [1]. Among the engineered nanomaterial, titanium dioxide (TiO_2_) nanoparticles are one of the most highly manufactured in the world and are widely used in paints, printing ink, paper, cosmetics, pharmaceuticals, sunscreen, bio-medical ceramic and implanted biomaterials, industrial photocatalytic processes and decomposing organic matters in wastewater [2–5]. Concerns regarding the potential health risks of these nanoparticles have been raised due to their inherent physicochemical attributes such as small size, increased surface area, conductivity and aggregation potential. Studies on the bio-distribution of TiO_2_ nanoparticles have indicated the liver as one of the principal sites in the body for accumulation through intentional ingestion or indirectly through nanoparticle dissolution from food containers or secondary ingestion of inhaled particles [6, 7]. Additionally, increased environmental contamination and unintentional ingestion via water, food or animals may also result in subsequent accumulation of nanoparticles in the liver [8–10]. The concern about adverse health effects of low-level exposure to TiO_2_ is imperative to address, particularly to analyze whether TiO_2_ exposure causes damage to mitochondrial bioenergetics and the liver. Although there is a plethora of published literature on acute TiO_2_ toxicity, the effect of TiO_2_ exposure on the hepatocyte mitochondria and its implications on the liver biology remains to be investigated. The current knowledge in the field of hepatotoxic effects of TiO_2_ nanoparticles is not yet exhaustive, and further investigation is necessary to fully elucidate the pathogenesis of the liver damage and the potential relationship between liver toxicity and the different characteristics of nanoparticles. Interestingly, the interactions between TiO_2_ nanoparticles and DNA, both direct and indirect, such as those mediated by oxidative stress, deserve greater attention in order to understand their potential role in the mechanisms underlining genotoxic and carcinogenic effects.

The liver is a multicellular organ that performs numerous vital metabolic, synthetic and clearance-related functions in mammals [11]. Hepatocytes account for approximately 80% of the liver mass and exhibit high metabolic and biotransforming activity that consequently imposes high energy requirements and is regulated by the high density of mitochondria, distributed uniformly throughout the cell body [12, 13]. Mitochondria acts as the vital source of energy in hepatocytes and also play an important role in extensive oxidative metabolism and normal functioning of the liver [14]. Inherently, mitochondria have an extremely dynamic nature; they undergo continual fission and fusion processes that counterbalance each other, to alter the organelle morphology that enables the cell to meet its metabolic requirements and cope with internal or external stress [15, 16]. Three central players that control the process of mitochondrial fission and fusion resulting in the unique structural features have been identified in mammals: (1) Mitofusins 1 and 2 (Mfn-1 and Mfn-2); for outer-membrane fusion (2) OPA1; for inner membrane fusion and (3) Drp1 for inner and outer membrane fission [16]. In normal conditions, mitochondrial fusion enhances mitochondrial integrity by allowing component sharing across the tubular network. However, the fusion of highly damaged mitochondria to the network could be detrimental since impaired mitochondria generate reactive oxygen species (ROS) that results in substantial cellular damage [14, 16]. Numerous environmental factors can also lead to excess ROS production and oxidative stress. Damage to mitochondrial dynamics and biology has been demonstrated to be a vital factor in several liver disorders [12, 17–21]. Functional impairment of mitochondria in hepatocytes due to oxidative stress is often accompanied by modification of mitochondrial proteins, DNA and lipid peroxidation which may lead to mitochondrial bioenergetics failure, that eventually leads to compromise in cellular functions and subsequent necrotic or apoptotic cell death [22]. Diminished OPA1 and Mfn (1 and 2) levels have been reported in biological systems that are in a diseased state [23, 24]. Sebastian et al. reported that a liver-specific knockout of Mfn-2 protein resulted in disrupted glucose metabolism in the liver, forming a potential cause for type II diabetes [25]. Recent studies have demonstrated that exposure to several engineered materials, including nanomaterials, leads to structural and functional alterations in mitochondrial membranes [26, 27]. Thus, studies on understanding the effect of nanoparticles exposure on liver function and mitochondrial biology is the need of the hour in order to address the implications of nanoparticles exposure on potential liver diseases but is very limited in the literature.

In this study, we investigated the perturbations in the liver behavior and mitochondrial characteristics caused by exposure to TiO_2_ nanoparticles on primary hepatocytes isolated from rat liver. We utilized three commercially employed TiO_2_ nanoparticles (P25, Anatase, and Rutile), to investigate nanoparticle specific perturbation in an explicit range of concentrations mimicking TiO_2_ nanoparticle accumulation. Additionally, we evaluated the effect of TiO_2_ nanoparticles exposure on mitochondrial health and oxidative stress as indicators of perturbations in normal liver function. These findings are the first step towards broadening our understanding on the molecular mechanisms of liver dysfunction induced by these highly utilized nanoparticles. Our findings also demonstrate detrimental effects of TiO_2_ nanoparticles on cellular and mitochondrial function in primary hepatocytes and suggest that mitochondrial stress can be used as an early and potent diagnostic marker for nanotoxicological inquiries in the liver.

## Materials and Methods

### Preparation of TiO_2_ nanoparticle suspensions

Degussa P25 (particle size 21 nm) was obtained from Sigma Aldrich, St. Louis, MO. Pure rutile (particle size 50 nm), and pure anatase (particle size 50 nm) were purchased from MK Nano, Mississauga, Ontario, Canada. The nanoparticles were UV sterilized and stock suspensions were made in sterile Phosphate Buffer Saline (PBS) at pH 7.4 and sonicated [FS30D Fisher Scientific] for 30 min and stored in dark at 4°C until use.

### Isolation, Culture and Treatment of Primary Hepatocytes

All the animal procedures were carried out in accordance with the guidelines from IACUC of University of Nebraska-Lincoln. Primary rat hepatocytes were isolated from male Sprague-Dawley rats weighing 160-200g through a two-step collagenase perfusion technique adapted from P.O Seglen [28]. Around 150–200 million cells were obtained at a viability greater than 85% as confirmed by Trypan blue dye exclusion test. Before seeding, tissue culture plate surfaces were coated with 100 μg/ml rat tail collagen type I solution prepared in 0.02N acetic acid for 1 hour at 37°C, washed and stored at 4°C until use. Cells were seeded at a density of 100,000/cm^2^ on the collagen coated plates. Nanoparticle suspensions in the desired concentrations were prepared in the culture media and added to the cells.

### Primary Hepatocyte Culture medium

Culture medium was prepared with high glucose DMEM supplemented with 10% fetal bovine serum, 0.5 U/ml insulin, 20 ng/ml epidermal growth factor (EGF), 7 ng/ml glucagon, 7.5 mg/ml hydrocortisone, and 1% penicillin-streptomycin. All the constituents for the cell culture medium was obtained from Sigma Aldrich, USA.

### Dynamic Light Scattering Particle Sizing and Zeta Potential Measurement

TiO_2_ Nanoparticle size and zeta potential were measured using a NanoBrook ZetaPALS zeta potential and dynamic light scattering instrument [Brookhaven instrument, Holtsville, NY]. Desired concentrations of nanoparticle suspensions were prepared by dilution with Hepatocyte culture medium. Mean hydrodynamic diameter was measured at a scattering angle of 90°, and the Zeta potential was calculated from Mobility measurements by using the Smoluchowski model. All measurements were performed at 25°C at a pH of 7.4.

### Scanning Electron Microscopy

Nanoparticle size and shape were assessed and viewed under a scanning electron microscope (SEM) [S-3000N, Hitachi Tokyo, Japan]. Cellular morphology and nanoparticle distribution were visualized by SEM. The cells were rinsed with PBS and fixed with 4% paraformaldehyde/PBS solution for 15 min. The paraformaldehyde solution was removed, samples rinsed with PBS and dehydrated with ethanol solutions (from 20 to 100%). The sample was incubated for 15 min at room temperature in each solution. The 100% ethanol solution was removed with hexamethyl disilazane [Sigma Aldrich, USA], and the sample was allowed to air-dry. The samples were then coated with gold-palladium (Au-Pd) and analyzed under the SEM.

### Lethal Concentration (LC_50_) Assessment using MTT Assay

The cytotoxicity of nanoparticles was assessed by MTT assay [3-(4,5-dimethyldiazol-2-yl)2,5 diphenyl Tetrazolium Bromide] [Life Technologies, NY] which quantitatively evaluates the mitochondrial conversion of the MTT salt into purple formazan crystals. Nanoparticle solution was removed, and 0.5 mg/ml MTT working solution in DMEM was incubated on live cells at 37°C for 2.5 h. After incubation, the working solution was removed, and lysis buffer (0.1 N HCl in Isopropanol) added. The lysis buffer was transferred to a 96 well plate and absorbance values collected in an AD340 plate reader [Beckman Coulter, Brea, CA] at corrected 570/620 nm. Relative absorbance was used as the indicator of cell viability. Concentration range of 0 ppm to 1000 ppm for each nanoparticle was used to generate the dose-response curve. SigmaPlot software was used to calculate LC_50_ value for each type of nanoparticle. Data were expressed as the means ± SD from three independent experiments.

### Urea Assay

Urea secretion by hepatocytes in culture medium was assessed every 24 h using Stanbio Urea Nitrogen (BUN) kit [Stanbio, Boerne, TX] using manufacturer’s protocol. Briefly, the kit exploits the reaction between urea and diacetyl monoxime which results in a color change at an absorbance of 520 nm read on AD 340 plate spectrophotometer [Beckman Coulter, Brea, CA]. Data were expressed as the means ± SD from six independent experiments.

### Albumin ELISA

Albumin Secretion by hepatocytes into culture medium was measured every 24 h using Rat Albumin ELISA Quantitation Kit from Bethyl Laboratories, Inc [Montgomery, TX] according to manufacturer’s instructions. In short, a 96 well plate was coated with a coating antibody for 1 hour and blocked with BSA for 30 min. Standard/Sample was added to each well and incubated for 1 hour. HRP detection antibody was incubated for 1 hour, followed by the addition of TMB Substrate solution that was developed in the dark for 15 min. Absorbance was read on AD340 plate spectrophotometer [Beckman Coulter, Brea, CA] at 450 nm. Data were expressed as the means ± SD from six independent experiments.

### Live/Dead Assay

Cell viability was assessed using a Live/Dead Viability/Cytotoxicity Kit [L-3224 Invitrogen, Grand Island, NY]. Briefly, post-treatment, primary hepatocytes were washed with PBS and incubated at 37°C for 30 min with assay reagent (4 μM EthD-1 and 2 μM Calcein in PBS) at 37°C. The cells were removed and washed three times with PBS and viewed with an Axiovert 40 CFL [Zeiss, Germany] and X-Cite 120Q [Lumin Dynamics, Mississauga, Ontario, Canada].

### Transmission Electron Microscopy (TEM)

Stable suspensions of the different nanoparticles were prepared in DI water using sonication. The samples were prepared for imaging by sequential drying steps on copper grids [Ted Pella Inc., CA] that were coated with carbon. Hitachi H7500 TEM was used for analyzing the samples.

### Reactive Oxygen Species (ROS) Quantification

Reactive Oxygen Species (ROS) production was quantified by an H_2_DCFDA based fluorescence assay. Briefly, the cells were washed to remove traces of serum from the culture media and were incubated with 10μM H_2_DCFDA [Life Technologies, NY] for a duration of 30 min at 37°C. After incubation, cells were gently washed, and cells were trypsinized using TRYPLE select [Life Technologies, NY] and suspended in PBS. The cell suspension was transferred to a 96 well plate, which was read at excitation 528 nm and emission 405 nm using a SLFA plate reader [Biotek, Winooski, VT]. Hydrogen Peroxide treatment was used as a positive control, and the untreated hepatocytes were used as the experimental control to normalize the fluorescence intensity. Data were expressed as the means ± SD from four independent experiments. Each experiment was carried out with three experimental replicates.

### Gene Expression

At each time point, total RNA from primary hepatocytes was isolated using RNeasy Micro Kit [Qiagen, Valencia, CA] according to the manufacturer’s instructions. Briefly, cells were trypsinized, centrifuge pelleted, washed with PBS and lysed in RLT buffer with equal volume 70% ethanol. The mix was then centrifuged in an RNeasy spin column, washed and concentrated until the final RNA was released into RNase-free water. The quality and quantity was determined by ND-1000 spectrophotometer [NanoDrop Technologies Wilmington, DE]. Equal amount of total RNA (1 μg) from each sample (treated and untreated) was reverse transcribed using iScript cDNA synthesis kit [Bio-Rad Laboratories, CA] by following manufacturer’s instructions.

Quantitative Real-Time PCR was performed using SYBR Green Master Mix [Applied Biosystems, Foster City, CA] in an epgradient S Mastercycler [Eppendorf, NY]. The primers of interest were obtained from Integrated DNA Technologies [Coralville, IA] with the following sequences: OPA-1 (Forward 5’- CCTGTGAAGTCTGCCAATCC -3' and Reverse 5’- CTGGAAGATGGTGATGGGTT -3'), Mfn1 (Forward 5’-TCGTGCTGGCAAAGAAGG-3’ and Reverse 5’-CGATCAAGTTCCGGGTTCC-3’). GAPDH (Forward 5’ ATGATTCTACCCACGGCAAG 3’ and Reverse 5’ CTGGAAGATGGTGATGGGTT 3’) was used as the housekeeping gene. A single PCR product formation was monitored using the SYBR green compatible melting curve analysis. Double normalization was carried out with respect to total RNA and the housekeeping gene and the relative gene expression levels of the target genes were reported using the ΔΔCT method of analysis. Data were expressed as the means ± SD from three independent experiments. Each experiment was carried out with three experimental replicates.

### Mitochondrial Morphology Imaging

Mitotracker FM, green stain [Life Technologies, NY] was used for the specific staining of primary hepatocyte mitochondria. Live cells were washed with PBS, and the dye was diluted to a concentration of 100 nM in Fluorobrite DMEM [Life Technologies, NY] and added to the cells. Cells were incubated at 37°C for 45 min and then washed extensively and imaged using confocal microscopy (Olympus FV500 IX 81).

### Mitochondrial Membrane Potential (MMP) Assay

MMP of primary hepatocytes was determined using Tetramethylrhodamine (TMRM) [Life Technologies, NY] staining. TMRM is a cationic dye that selectively stains healthy mitochondria that are depolarized. A working solution of concentration 50 nM was prepared in Fluorobrite DMEM and added to the cells. The cells were incubated in the dark at room temperature for 45 minutes, following which, the cells were washed 3X with PBS. Cells were trypsinized using TRYPLE select [Life technologies, NY] and resuspended in Fluorobrite DMEM. The fluorescence of the cells was recorded through flow cytometry using a FACSCantoll from Becton Dickenson [Franklin Lakes, NJ]. The cell suspensions were transferred to flow cytometry tubes and samples were analyzed for fluorescence in the red channel (excitation 573 nm and emission 590 nm). The fold change in the cell staining for the untreated and treated cells, relative to the unstained hepatocytes, was reported. Data were expressed as the means ± SD from three independent experiments.

### Manganese Superoxide Dismutase (MnSOD) Enzyme Activity Assay (In Gel)

MnSOD enzyme activity of primary hepatocytes was measured using a gel assay. Protein was collected from cells post treatment using RIPA buffer with PMSF and protease inhibitors. Total protein quantity was determined using Bradford assay. Total of 30 μg protein was loaded onto 10% native Tris-Glycine polyacrylamide gels, and polyacrylamide gel electrophoresis (PAGE) was carried out in non-denaturing conditions to ensure intact MnSOD enzyme activity. The gel was then incubated in the dark in a staining solution containing 0.1 mg/ml riboflavin, 0.1mg/ml nitroblue tetrazolium (NBT) and 1μl/ml TEMED. The gel was washed with DI water and exposed to light. The superoxide released by TEMED interacts with NBT converting it into purple formazan. This turns the gel purple except in the area of the gel with MnSOD, which scavenges the superoxide giving rise to colorless bands. The gels were analyzed using the imaging system of Odyssey by LI-COR followed by the imaging software Image Studio. Data were expressed as the means ± SD from three independent experiments.

### Statistical Analysis

Data were expressed as the mean ± SD from three independent experiments. The difference between the various experimental groups was analyzed by a one-way analysis of variance (ANOVA) using the statistical analysis embedded in SigmaPlot Software using Tukey test. Q tests were employed to identify outliers in the data subsets. For statistical analysis of all data, p < 0.05 was used as the threshold for significance.

## Results

### TiO_2_ Nanoparticle Characterization

**Fig 1:**
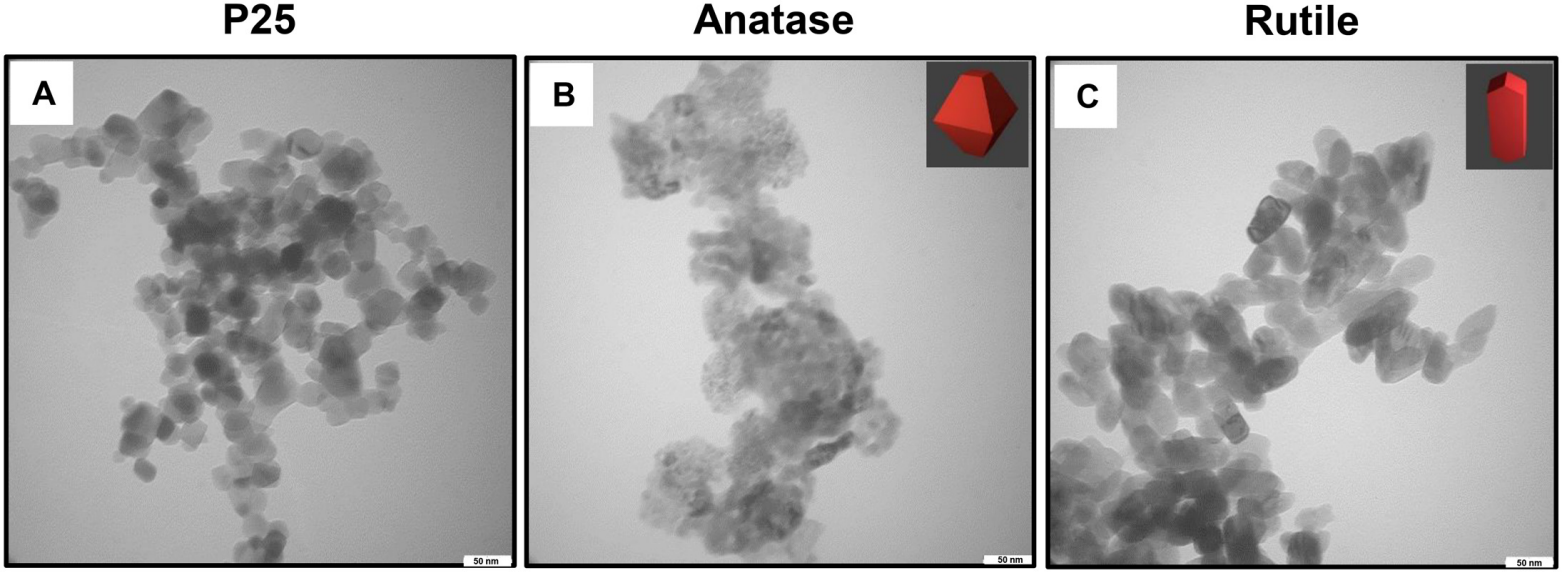
Transmission Electron Microscopy images to characterize the crystal shape of the TiO_2_ nanoparticles as seen in DI water; (a) P25, (b) Anatase, 50 nm particle size and (c) Rutile, 50 nm particle size.

TiO_2_ nanoparticles were first characterized using TEM and DLS. TEM was utilized to examine the individual crystal shapes and sizes of the different TiO_2_ nanoparticles (Fig 1). Anatase TiO_2_ nanoparticles revealed the characteristic spherical crystal structure, and rutile nanoparticles displayed a typical rod-like crystal structure. Both particles displayed a size of approximately 50 nm. P25, which is a 3:1 mixture of anatase and rutile. These results were in agreement with the manufacturer’s specifications and previous reports on the characterization of the shape of the nanoparticles.[6, 29, 30].

The nanoparticle suspensions were then characterized for the hydrodynamic diameter of the aggregates formed and the zeta potential in the media environment that is exposed to the cells using DLS. The working concentration of nanoparticles suspensions were prepared in hepatocyte media to recreate the cell culture conditions to identify the forms in which the nanoparticles are exposed to the cells. As shown in Table 1, P25, anatase, and rutile nanoparticles aggregated to average diameter of approximately 800 nm, 700 nm, and 380 nm, respectively, and this aggregation was consistent in all the studied nanoparticle concentrations. Zeta potential were also measured for the three TiO_2_ nanoparticles (Table 1). The zeta potential values did not change significantly (p > 0.05) in the three forms of the nanoparticles and concentrations.

**Table 1:** Characterization of TiO_2_ nanoparticles aggregates forming in hepatocyte culture medium using Dynamic Light Scattering (DLS) at 37°C and pH of 7.4.

TiO_2_ Nanoparticle	Concentration (ppm)	Hydrodynamic Diameter (nm)	Polydispersity Index	Zeta Potential (mV)
**P 25**	20	841.7 ± 85.3	0.279	-10.8 ± 2.5
	50	783.5 ± 85.3	0.157	-8.7 ± 3.5
	100	784.1 ± 54.5	0.226	-6.4 ± 2.4
**Anatase**	20	739.1 ± 86.9	0.232	-8.7 ± 4.3
	50	659.2 ± 35.0	0.166	-7.4 ± 2.8
	100	692.4 ± 59.4	0.178	-9.9 ± 4.3
**Rutile**	20	380.0 ± 29.1	0.236	-10.2 ± 1.8
	50	374.8 ± 34.5	0.189	-8.5 ± 4.6
	100	396.3 ± 13.5	0.248	-6.5 ± 4.3

### TiO_2_ Nanoparticles Cytotoxicity to Primary Hepatocytes

We evaluated the cytotoxicity of three different TiO_2_ nanoparticles (P25, anatase, and rutile) using MTT assay. A 72 h exposure to the three different TiO_2_ nanoparticles of varying concentration (0–1000 ppm) to primary hepatocytes established the LC_50_ value corresponding to the different treatment, as determined by constructing a dose-response curve. As seen in Table 2, the LC_50_ values of P25, anatase and rutile TiO_2_ nanoparticles were 74.13±9.72 ppm, 58.35±4.76 ppm, and 106.81±11.24 ppm, respectively. S1 Fig represents the dose-response curves plotted for the different nanoparticles using non-linear regression.

**Table 2:** Lethal Concentration (LC_50_) analysis of the different TiO_2_ nanoparticles treatment of primary rat hepatocytes.

Nanoparticle Type	LC_50_ value (in ppm)
P 25	74.13 ± 9.72
Anatase	58.35 ± 4.76
Rutile	106.81 ±11.24

### TiO_2_ Nanoparticle and Hepatocytes Morphology

**Fig 2:**
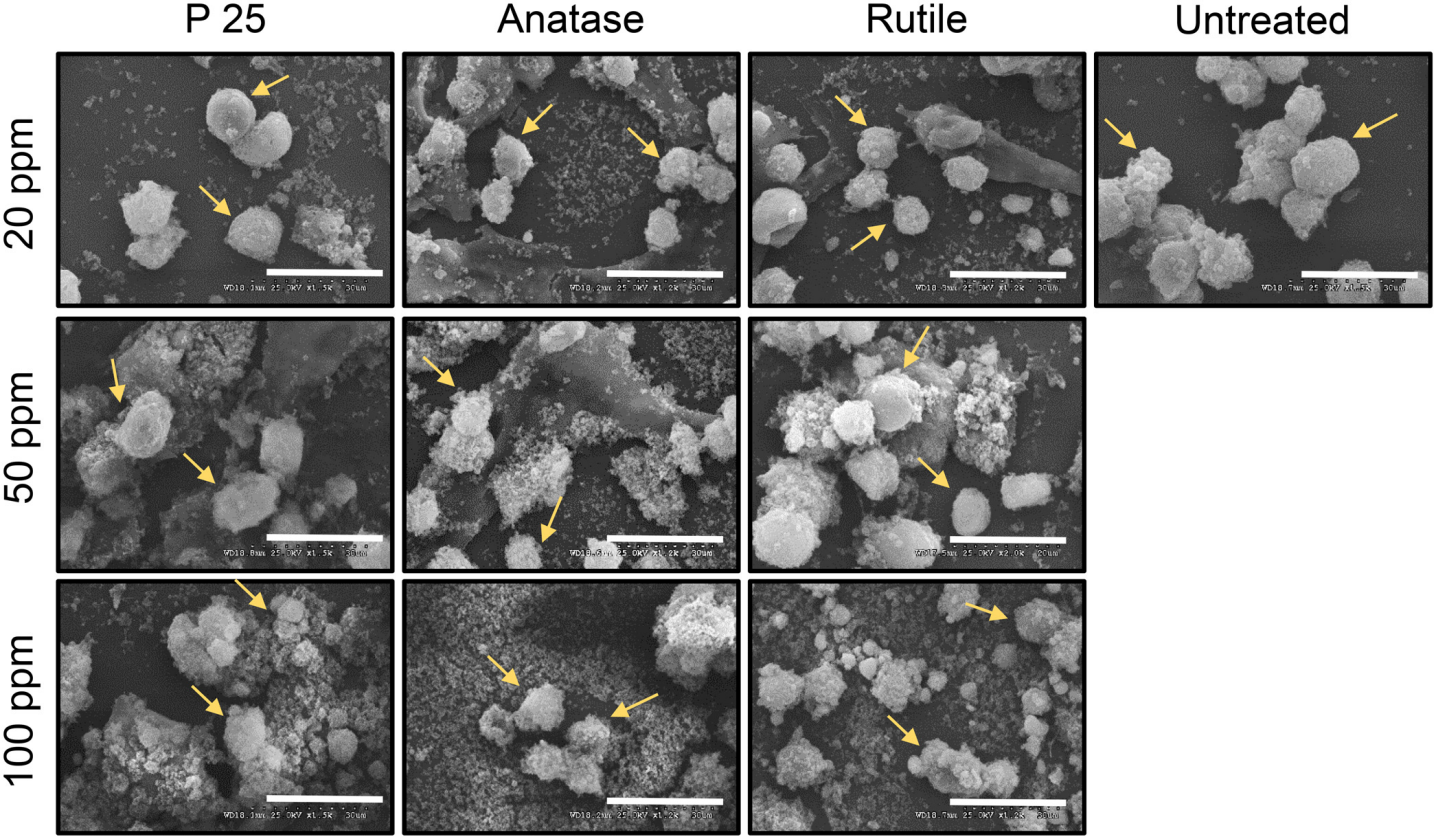
Scanning Electron Microscopy (SEM) images to visualize the morphology of primary hepatocytes when treated with TiO_2_ nanoparticles after 72 h of exposure. Scale bar: 30 microns. Yellow arrows point to primary hepatocytes.

We studied the effect of nanoparticle treatment on the cellular morphology using SEM (Fig 2). After 72 h of exposure to the three chosen concentrations of the nanoparticles, primary hepatocytes did not exhibit a marked change in cellular morphology. For all three nanoparticles, we observed the smooth and spherical morphology of hepatocytes that was comparable to untreated cells.

### TiO_2_ Nanoparticle and Hepatocytes Viability

**Fig 3:**
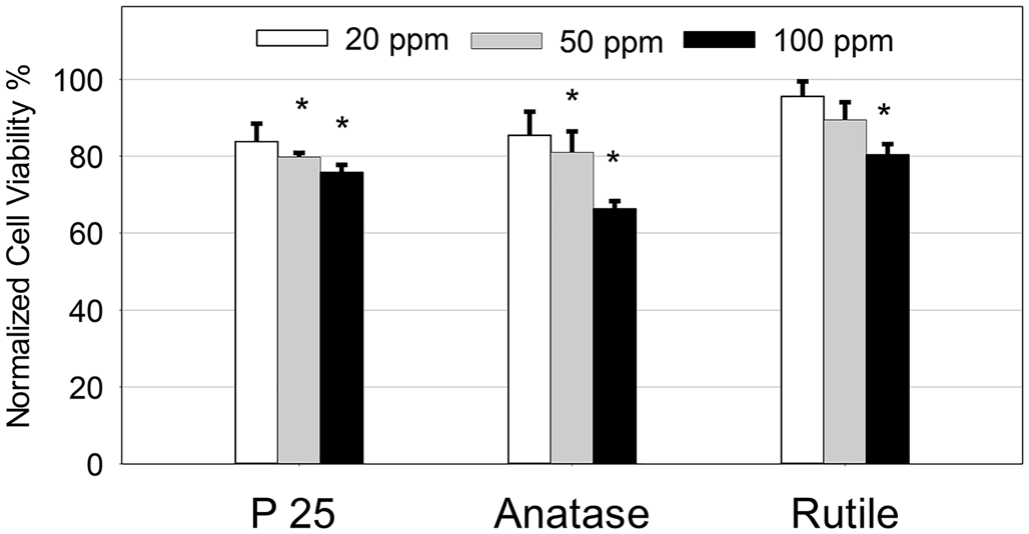
MTT assay to quantify primary hepatocyte viability after treatment with different TiO_2_ nanoparticles at 20, 50 and 100 ppm after 72 h of exposure normalized to the untreated hepatocytes. The values are the mean ± SD of five different samples, significant difference with respect to control is denoted as * p value < 0.001.

We quantified the viability loss in hepatocytes using MTT assay. The exposure of hepatocytes to TiO_2_ nanoparticles showed a concentration and type dependent loss in viability (Fig 3). When normalized with respect to untreated hepatocyte samples, in P25 treatment, 84% cells were viable when exposed to 20 ppm concentration that decreased to 75% at 100 ppm concentration. Similarly in hepatocytes exposed to anatase nanoparticles, the cell viability decreased substantially from 85% in the 20 ppm concentration to 66% in 100 ppm. In rutile treatment, the loss in viability was concentration dependent but demonstrated the least severity, where the cell viability was approximately 80% in the 100 ppm treated samples. In addition to the MTT assay, S2 Fig also provides qualitative analysis of the loss in cell viability in primary hepatocytes when exposed to the different nanoparticles.

### TiO_2_ Nanoparticle and Loss in Hepatocyte Functions

**Fig 4:**
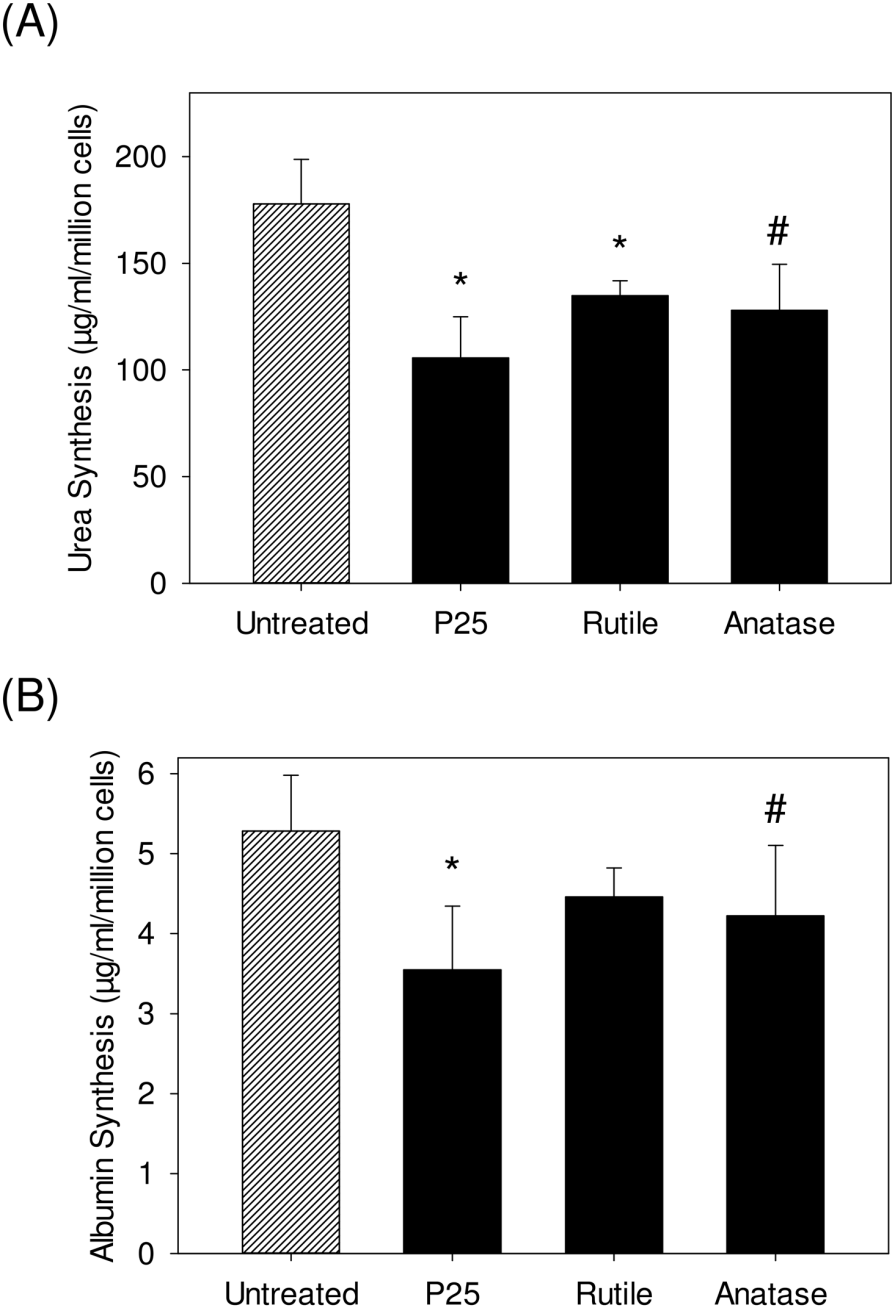
Characterizing the effect of the different TiO_2_ nanoparticles treatment (50 ppm) on primary hepatocytes specific functions (A) Quantification of urea synthesized primary hepatocytes after 72 h of exposure normalized to the untreated cells and (B) Quantification of albumin synthesized by primary hepatocytes after 72 h of exposure normalized to the untreated cells. The values are normalized with respect to loss in cell viability. The values are the mean ± SD of six different samples, significant difference with respect to control is denoted as * p value < 0.001, # p value< 0.05.

We studied the effect of prolonged exposure of hepatocytes to TiO_2_ nanoparticles on two main hepatocyte specific functions- urea synthesis and albumin synthesis. As seen in Fig 4A, we observed a consistent concentration and type dependent loss in urea synthesis function. For every million hepatocytes, the exposure of hepatocytes to 50 ppm of P25 resulted in 105.6±19 μg/ml urea synthesis, as opposed to 178 ± 20.9 μg/ml synthesis in untreated hepatocytes. Similarly, the exposure of hepatocytes to 50 ppm of anatase resulted in 127.9±21.6 μg/ml urea synthesis. Finally, the exposure of hepatocytes to rutile resulted in 134.7±6.9 μg/ml urea synthesis, which was relatively higher as compared to the 50 ppm treatment group of other particles.

Similarly, Fig 4B illustrates the albumin synthesis of primary hepatocytes after 72 h of exposure to different TiO_2_ nanoparticles. We observed a concentration and type dependent loss in albumin synthesis comparable to our data on urea production. For each million hepatocytes, the exposure to 50 ppm of P25 resulted in 3.5±0.8 μg/ml albumin production, as compared to untreated hepatocytes that synthesized 5.3±0.69 μg/ml albumin. The exposure of hepatocytes to 50 ppm of anatase resulted in 4.22±0.8 μg/ml albumin production. Finally, in the case of rutile treatment, the exposure of hepatocytes to 50 ppm resulted in 4.5±0.3 μg/ml albumin production. The comprehensive quantification of urea synthesis and albumin synthesis by hepatocytes cultured for a week demonstrated similar trend when exposed to the different concentrations of the TiO_2_ nanoparticles (S3 Fig).

### TiO_2_ Nanoparticle Effect on Oxidative stress and Mitochondrial Dynamics

**Fig 5:**
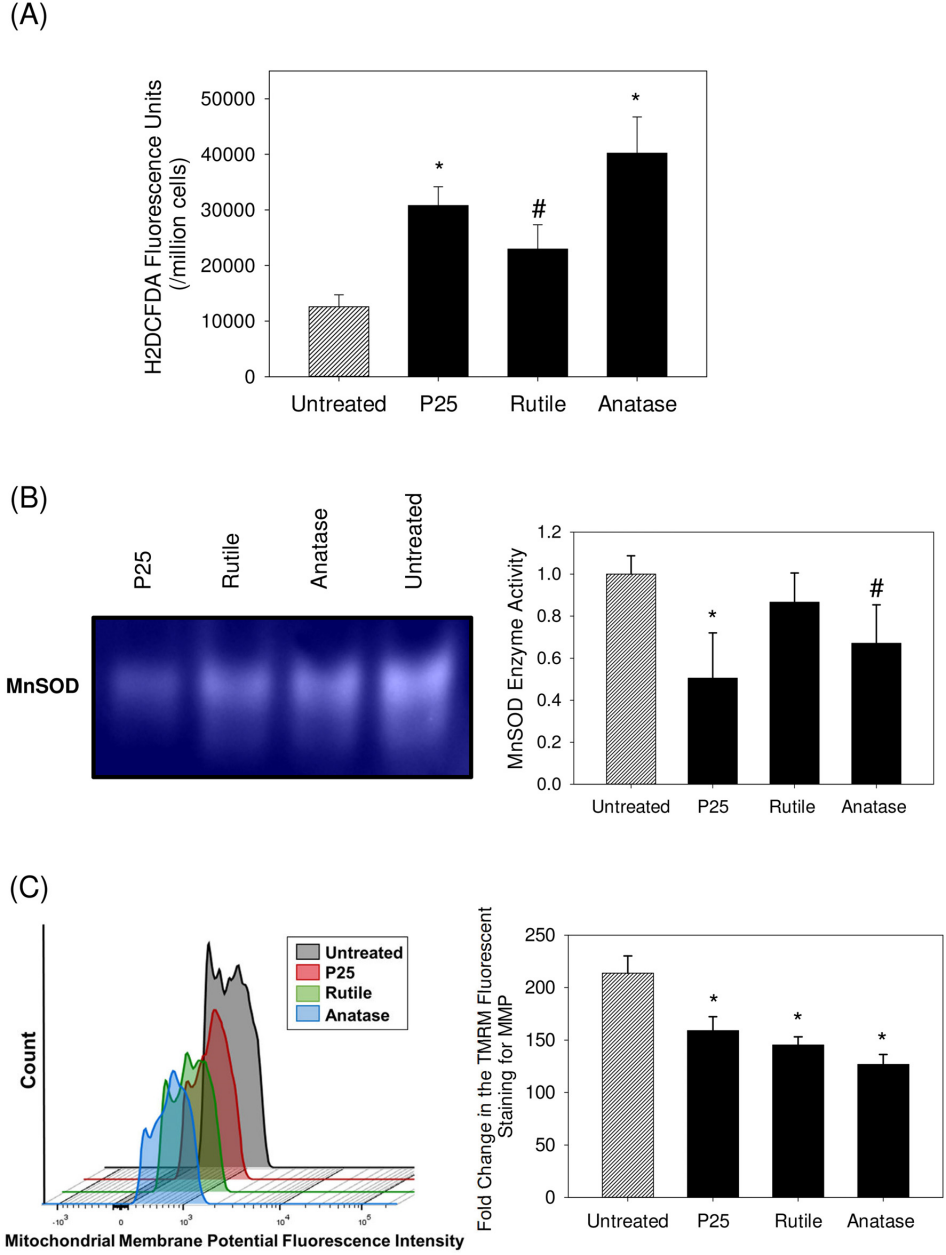
Characterizing the state of oxidative stress in primary hepatocytes upon TiO_2_ nanoparticle treatment at a concentration of 50 ppm for a duration of 72 h (A) Quantification of Reactive Oxygen Species produced using H_2_DCFDA based fluorescence assay (B) In gel mitochondrial MnSOD enzyme activity assay normalized with respect to untreated cells (C) Fold change in the TMRM staining to quantify mitochondrial membrane potential using flow cytometry reported relative to the unstained cells. The values are the mean ± SD of four different samples, significant difference with respect to control is denoted as * p value < 0.001, # p value< 0.05.

We quantified the ROS production using H_2_DCFDA dye in order to measure the increased oxidized status of the cells in response to nanoparticles exposure (Fig 5A). At a median concentration of 50 ppm, a type dependent increase in ROS production was observed when primary hepatocytes were exposed to TiO_2_ nanoparticles. The exposure of hepatocytes to 50 ppm of P25 and anatase resulted in relatively highest ROS production while exposure to the same concentration of rutile demonstrated lesser ROS production.

We studied the effect of TiO_2_ nanoparticle treatment on mitochondrial MnSOD enzyme activity (Fig 5B) and observed that the enzyme activity significantly decreased in each of the treatment groups (P < 0.05). As compared to untreated hepatocytes at 100%, P25 treated samples displayed 50.5±20.1% enzyme activity, followed by anatase at 67±18.2% and rutile at 86±13.8% enzyme activity. We also probed for the effect of the nanoparticle treatment on the MMP of hepatocytes (Fig 5C) and observed that the treatment leads to significant loss in MMP, as compared to untreated cells (p < 0.05).

### TiO_2_ Nanoparticle Effect on Mitochondrial Dynamics

**Fig 6:**
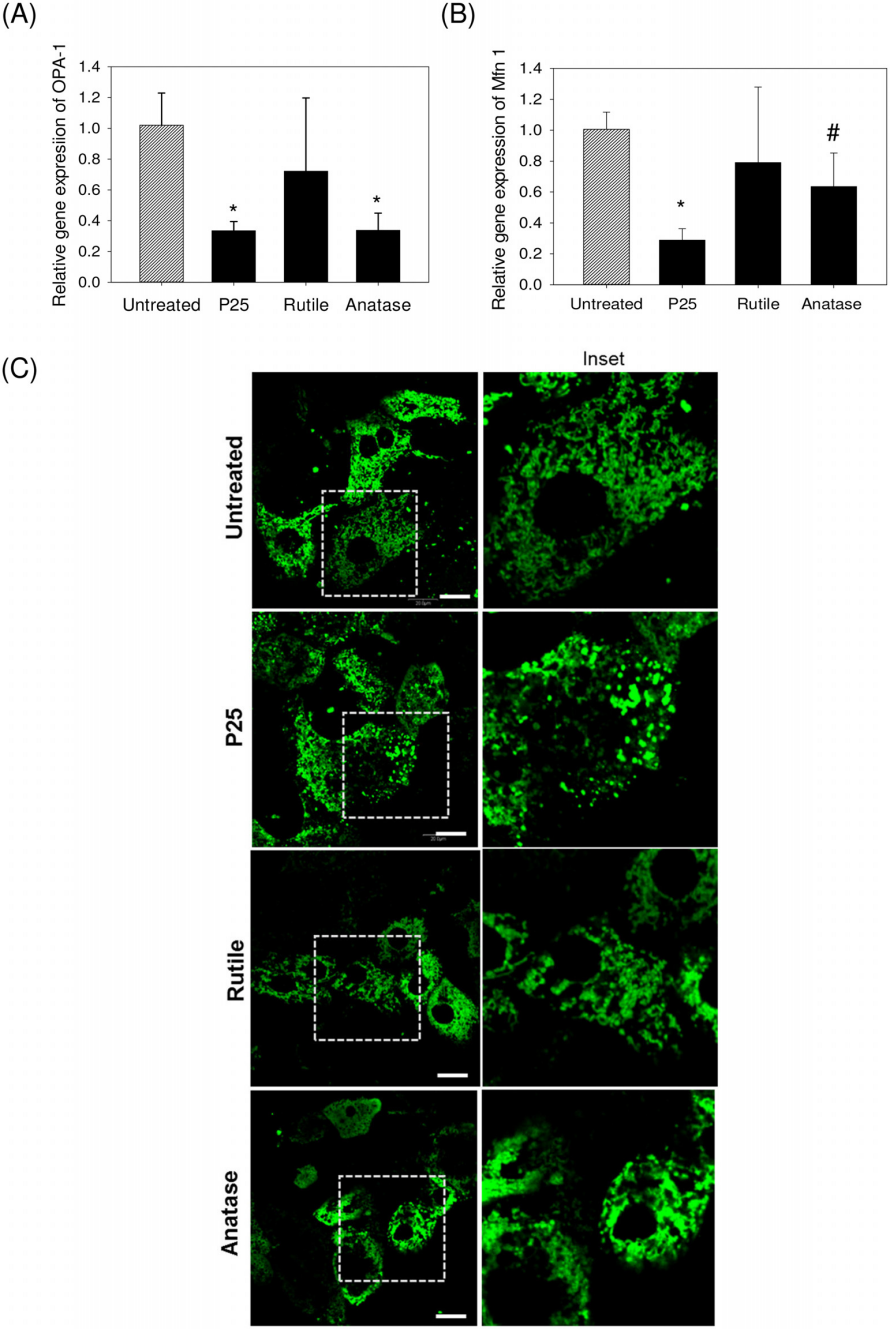
Characterization of the effect of TiO2 nanoparticle treatment (50 ppm) for duration of 72 h on primary hepatocyte mitochondrial dynamics. (A-B) Relative gene expressions of mitochondrial fusion markers through qPCR using double normalization with respect to total RNA and housekeeping gene (GAPDH). The values are the mean ± SD of four different samples, significant difference with respect to control is denoted as * p value < 0.001, # p value< 0.05. (C) Fluorescent imaging of the mitochondrial morphology in primary rat hepatocytes using Mitotracker green FM. Scale 20 microns. In the control image, long fiber-like mitochondrial morphology can be observed, as compared to fragmented and swollen mitochondria as seen in nanoparticle treated samples.

To understand the effect of nanoparticle treatment on mitochondrial dynamics, we investigated the relative gene expressions of OPA-1 and Mfn-1 markers that are associated with mitochondrial fusion events (Fig 6A and 6B). OPA-1 and Mfn-1 gene expression levels were significantly down-regulated in hepatocytes when exposed to 50 ppm P25 and anatase with commercially used P25 having the highest effect (p < 0.05). Down-regulation of the fusion markers in rutile treatment group was the least pronounced, as compared to anatase and P25. To probe and visualize the effect of the nanoparticles on the mitochondrial morphology and integrity, we imaged the mitochondria using the fluorescent stain Mitotracker FM (Fig 6). The untreated primary hepatocytes depicted the typical fiber-like morphology indicating healthy mitochondria. When hepatocytes were exposed to TiO_2_ nanoparticles, there was a substantial loss in the fiber-like morphology and presence of high levels of fragmentation was also observed.

## Discussion

The liver is the major accumulation site for many nanoparticles, however, the toxicological effects of the nanoparticles on the liver function have not been extensively investigated. Numerous *in vitro* liver models have been developed during the last two decades to supplement animal studies [31–38]. A major weakness of existing literature about the *in vitro* effects of nanoparticles is that the *in vivo* dosimetry and biokinetics are largely ignored, i.e., effects, if observed, are at high concentrations [39, 40]. A majority of the *in vitro* nanoparticle liver toxicity studies have extensively used cell lines. Several studies demonstrate that primary hepatocytes are a better *in vitro* model compared to cell lines such as HepG2 cells for cytotoxicity studies due to the inherent differences in the bio-transformation potential of cell lines vs. primary cells. Wang and co-workers demonstrated that hepatic cell lines depict different behavior with respect to metabolism mediated liver toxicants when compared to primary hepatocytes [41]. Harris and co-workers showed that primary hepatocytes are a preferred model when studying genotoxicity or carcinogenicity because the cell lines do not represent the exact genome of the target tissue they are modeling [42]. Studies have utilized liver-specific cell lines to demonstrate cytotoxicity effects of nanoparticles on liver [43, 44]. However, these studies are limited to common cytotoxicity end points such as MMP, glutathione, ROS and lactate dehydrogenase and do not address the impact of nanoparticle exposure on liver-specific functions such as urea or albumin synthesis and mitochondrial integrity. Our study utilizing primary hepatocytes provides a better *in vitro* model to study the impact of nanoparticle exposure on hepatocyte function and mitochondrial damage. Primary hepatocyte culture is a robust platform to study cytotoxicity effects in the liver compared to animal models. Xu and co-workers demonstrated a decrease in Blood Urea Nitrogen (BUN) levels (*in vivo* equivalent to urea synthesis) when mice were exposed to TiO_2_ nanoparticles [45]. Wang et al have also demonstrated a loss in mice liver functions through assessment of the liver enzymes and BUN when exposed to TiO_2_ nanoparticles [46]. Our studies utilizing primary hepatocytes demonstrate similar trends in liver specific functions when exposed to TiO_2_ nanoparticles as observed in animal studies. Our cytotoxicity studies indicated that pure anatase and P25 nanoparticles are more cytotoxic compared to rutile nanoparticles and this observation is consistent with previous reports comparing the anatase and rutile TiO_2_ nanoparticles [29].

The purpose of our study was to further probe the effect of TiO_2_ nanoparticles on primary hepatocytes focusing on the changes in cellular function and mitochondrial dynamics. Numerous studies have consistently used high concentrations of the nanoparticles, thus limiting these studies to probe mechanistic aspects beyond the toxicity of the nanoparticles. Sha et al demonstrated the effect of TiO_2_ nanoparticles on BRL-3A cell lines (concentrations of 0.1 to 100 μg/ml) and the liver of rat models (concentrations of 0.5–50 mg kg^−^1) where oxidative stress mediated toxicity was observed in both models [47]. Kermanizadeh and co-workers reported the occurrence of genotoxicity of TiO_2_ nanoparticles (0.5–256 μg/ml) in C3A cells [48]. Our study provided us with the range of concentrations (20, 50 and 100 ppm) with 72 h exposures that is reflective of the LC_50_ data. These concentrations fall in the sub-lethal range, thereby permitting us to investigate crucial early cellular events, which facilitated a better mechanistic understanding of the intrinsic factors mediating nanoparticle induced toxicity. Our results also indicated that there is a concentration and type dependent effect on primary hepatocytes when exposed to TiO_2_ nanoparticles. This difference in the cell behavior reflects on potentially different modes of actions from the different TiO_2_ nanoparticles on the hepatic biology. We also demonstrated a concentration and type dependent loss in urea and albumin synthesis function of hepatocytes. The most critical observation is the exposure to 100 ppm of commercially used P25 TiO_2_ nanoparticles for 72 h, though has 80% viable cells, results in an over 45% loss in hepatic functions. This indicated that employing cell viability as a sole marker for the effect of environment exposures including nanoparticles is a weak biomarker to identify potential risk factors of these exposures.

Numerous studies have demonstrated that metal oxide nanoparticle induced toxicity is primarily mediated by increased ROS production [49, 50]. We also demonstrated that TiO_2_ nanoparticle exposure in primary hepatocytes results in increased ROS production. We further showed that primary hepatocytes, when exposed to TiO_2_ nanoparticles, resulted in a loss in MnSOD enzyme activity and MMP. Cells possess a robust anti-oxidant mechanism to cope with and prevent the downstream damage from excess ROS. MnSOD scavenger enzymes are the first line of the antioxidant defense system protecting the cells from potential damage caused by excessive amounts of ROS by scavenging the superoxide radicals [51]. MnSOD is the prominent isomer of the enzyme that is abundant in the mitochondria and contributes to the maintenance of redox homeostasis inside mitochondria [51]. Our observation that the nanoparticle exposure significantly reduces the MnSOD enzyme activity is a strong indication that the antioxidant system is impaired, leading to potential irreversible damage to the cell, especially mitochondria [52]. We also observed significant loss in MMP in primary hepatocytes when exposed to all three nanoparticles. The maintenance of MMP in the mitochondria is critical for proper oxidative phosphorylation function to occur and is therefore considered a critical marker to evaluate mitochondrial perturbation [53, 54]. Decrease in the MMP leads to more ROS production in the mitochondria, thus contributing to further mitochondrial membrane damage. The fluctuations in MMP is considered as Tier 3 oxidative stress responses and can lead to apoptotic responses [53]. These data strengthens our hypothesis that exposure to nanoparticles results in substantial mitochondrial damage in primary hepatocytes and the increase in the ROS levels is not due to adaptive response.

Mitochondria are extremely dynamic in nature and undergo continual fission and fusion processes to alter the morphology that enables the cell to meet its metabolic requirements and cope with internal or external stress. OPA1 and Mfn-1 are markers known to be instrumental in regulating the fusion process in maintaining the mitochondrial dynamics. We observed a significant down-regulation in the gene expression levels of OPA1 and Mfn-1 in the 50 ppm treated hepatocytes, whereas, this down-regulation was not significant in the rutile treated cells (p > 0.05). The exposure to nanoparticles also resulted in a substantial loss in the fiber-like morphology and increase in fragmentation. Braydich-Stolle and co-workers showed similar effect due to TiO_2_ nanoparticle treatment on keratinocytes that resulted in localization and causative damage of the mitochondria [55]. Hepatocytes possess a unique mitochondrial organization wherein the mitochondria are spread throughout the cell body unlike other cells where the mitochondria are concentrated around the cell nuclei and concentration decreases radially. Loss in the typical fiber-like morphology and increase in fragmentation is a strong indication of compromise in the mitochondria dynamics. This is in agreement with our observation where OPA-1 and Mfn-1 were significantly downregulated in hepatocytes exposed to TiO_2_ nanoparticles. This defect in mitochondrial fusion results in mitochondria that appear swollen and spherical, instead of fiber-like. Together, these results indicate that exposure to TiO_2_ nanoparticles even at a concentration as low as 50 ppm results in significant mitochondrial damage by interrupting the fusion-fission equilibrium and affecting the mitochondrial dynamics [56].

**Fig 7:**
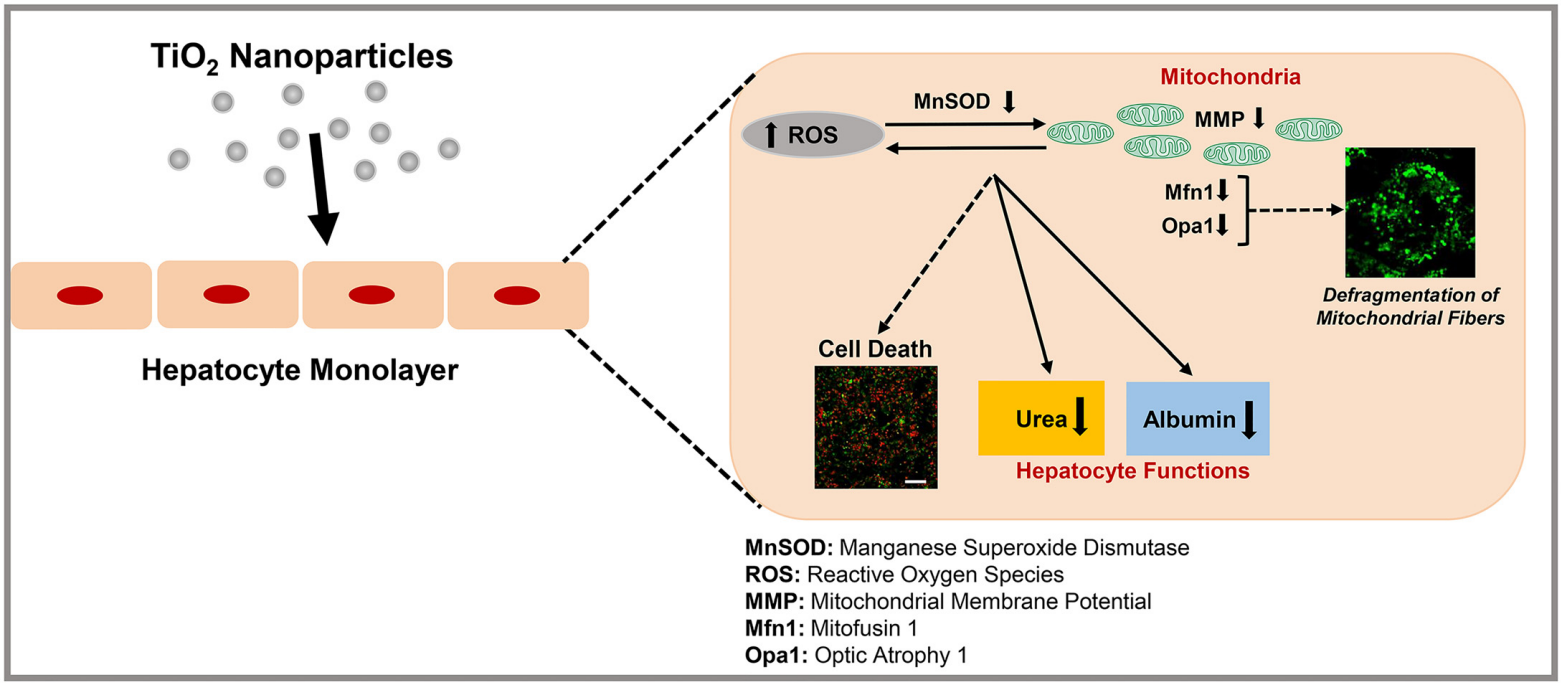
Schematic representation of the possible damaging role of TiO_2_ on primary hepatocytes. We propose that TiO_2_ induces loss in hepatocyte functions on primary hepatocytes through the induction of oxidative stress mediated by an increase of ROS production, loss in MnSOD enzyme function, loss in MMP and damage to mitochondria dynamics by down-regulating the fusion cycle in the mitochondrial dynamics.

Overall, we observed that the exposure of primary rat hepatocytes to different types of commercially available TiO_2_ nanoparticles causes significant compromise in hepatocyte function and mitochondrial biology. Even though we observed a modest loss in cell viability, hepatic specific functions, urea and albumin synthesis, are significantly reduced due to TiO_2_ nanoparticles exposure at concentrations as low as 50 ppm. We observed an increase in the amount of intracellular ROS production due to exposure to TiO_2_ nanoparticles. A decrease in the enzyme activity of MnSOD demonstrated compromise in the antioxidant defense mechanism and irreversible oxidative damage. Loss in mitochondrial membrane potential demonstrated the loss in mitochondrial oxidative phosphorylation function. Finally, we observed that the exposure to TiO_2_ nanoparticles resulted in significant down-regulation of OPA1 and Mfn-1 genes and fragmented mitochondrial network in hepatocytes that is a strong indicator of the disruption of the mitochondrial dynamics. From these observations, we propose that TiO_2_ nanoparticles induce cytotoxicity of hepatocytes by (1) down-regulating the fusion process thus disrupting the mitochondrial dynamics and inducing damage to the mitochondrial morphology, (2) triggering oxidative stress mediated by an increase in ROS production, loss in MMP and loss in MnSOD enzyme activity and (4) inducing loss in hepatic functions including urea and albumin (Fig 7). Therefore, we hypothesize that TiO_2_ nanoparticles could potentially contribute to subsequent adverse health effects and the development of liver diseases such as liver fibrosis. Future work is underway focusing on how these nanoparticle induced compromise of the mitochondrial dynamics in hepatocytes leads to liver damage and other potential liver diseases.

## Supporting Information

S1 Fig Dose response curve to calculate LC_50_ using four parameter plots for the different titanium dioxide nanoparticle treatment on primary hepatocytes. (TIF)

S2 Fig Live/Dead fluorescent staining of primary hepatocytes when treated with titanium dioxide nanoparticles on Day 7 in culture. Calcein FM stains the live cells green and Ethidium Bromide stains the dead cells red. Scale bar: 100 microns.
(TIF)

S3 Fig (A) Quantification of urea synthesized primary hepatocytes from day 1 to day 7 in culture when treated with the different TiO2 nanoparticles and (B) Quantification of albumin synthesized by primary hepatocytes when treated with the different TiO2 nanoparticles. All the data points are normalized to untreated hepatocytes.
(TIF)
