# Supplementary figures and images for: Titanium Dioxide Nanoparticles Trigger Loss of Function and Perturbation of Mitochondrial Dynamics in Primary Hepatocytes
Source: PLoS One. 2015 Aug 6;10(8):e0134541. doi: 10.1371/journal.pone.0134541 (PMC4527597; doi:10.1371/journal.pone.0134541)

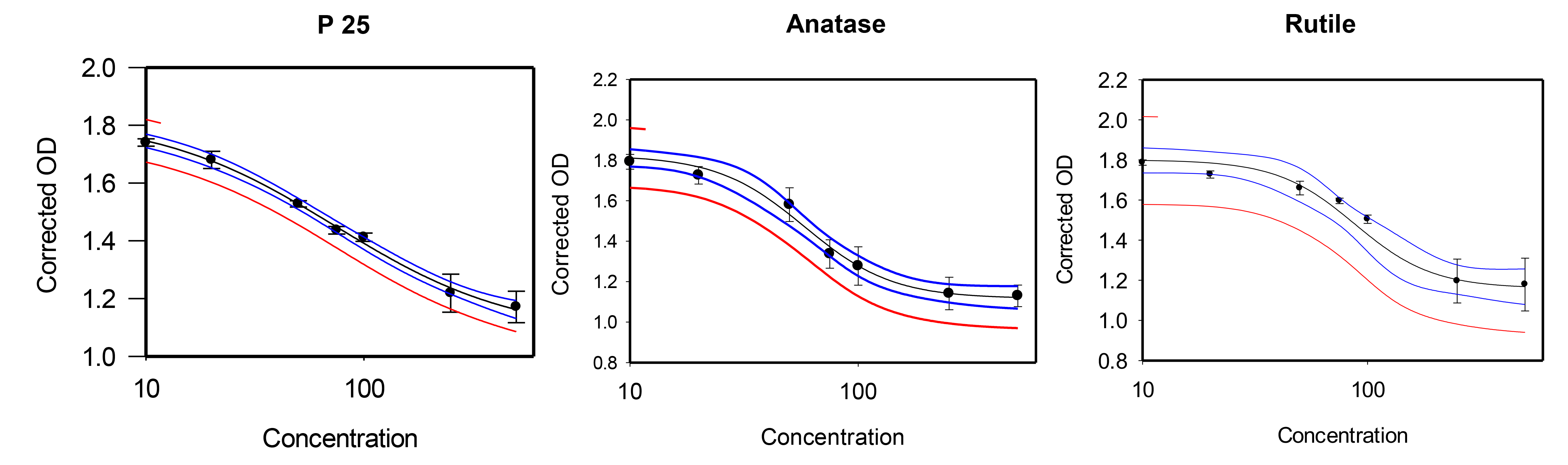

Supplement: S1 Fig — (TIF) [file pone.0134541.s001.tif]

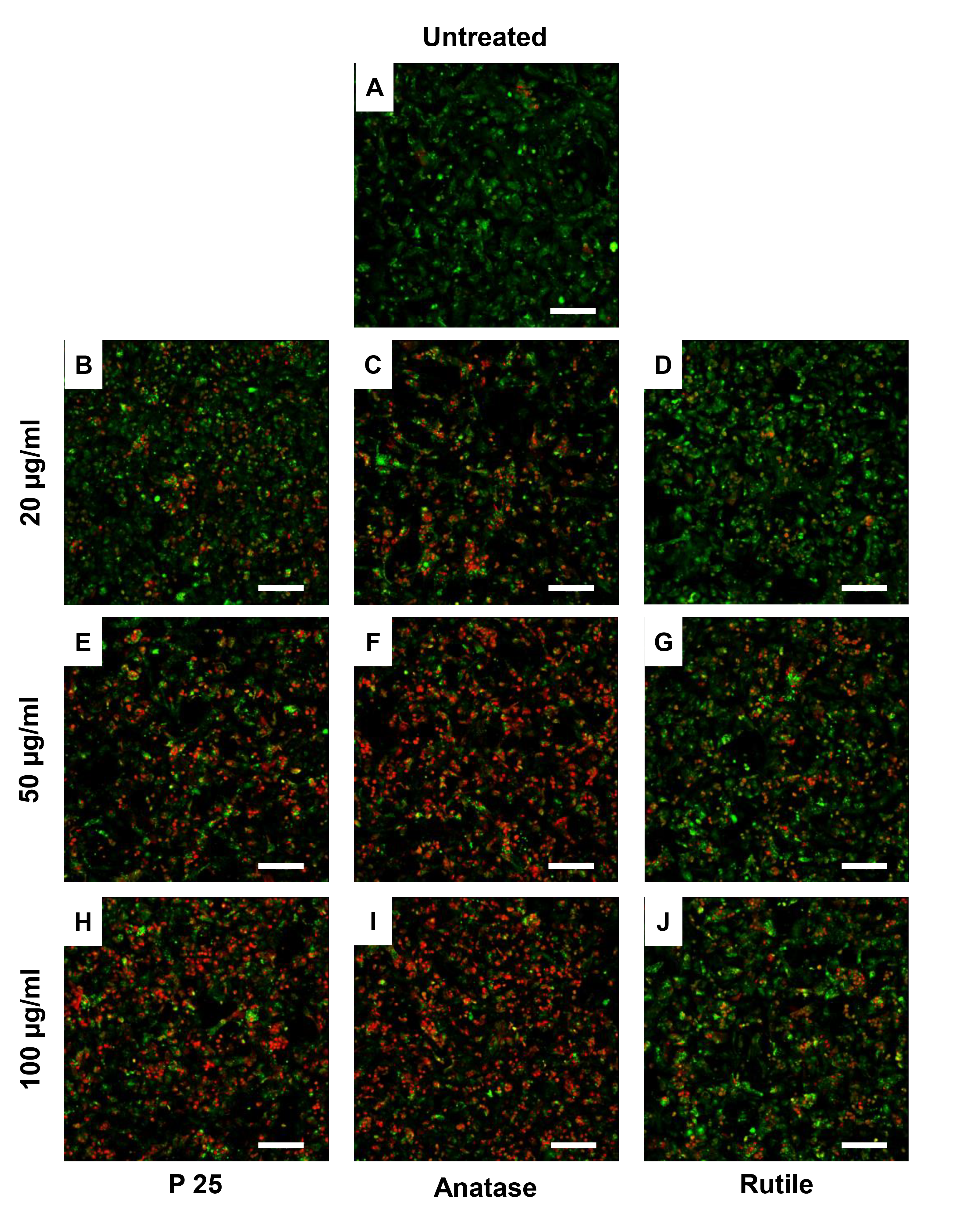

Supplement: S2 Fig — Calcein FM stains the live cells green and Ethidium Bromide stains the dead cells red. Scale bar: 100 microns. (TIF) [file pone.0134541.s002.tif]

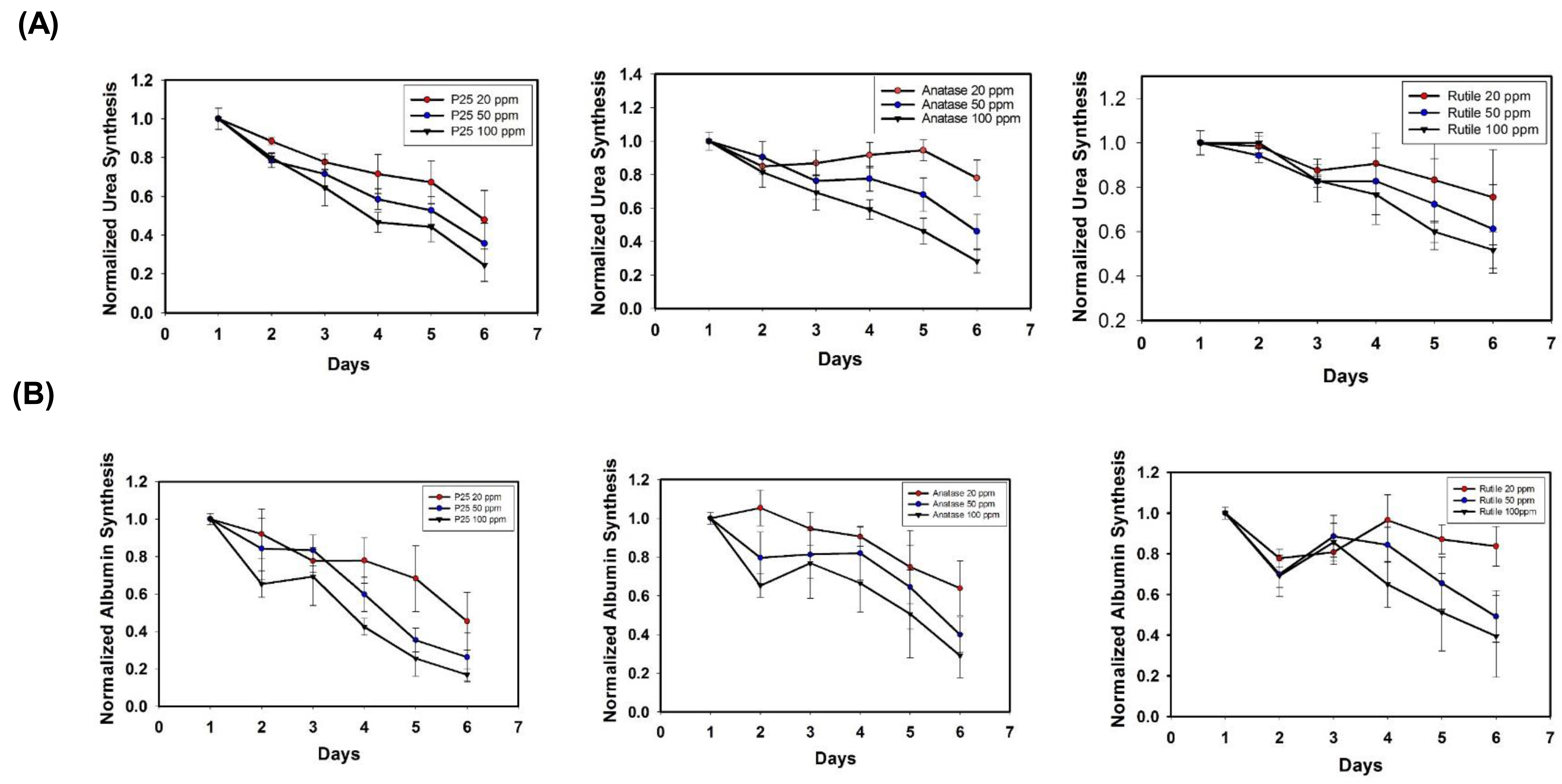

Supplement: S3 Fig — All the data points are normalized to untreated hepatocytes. (TIF) [file pone.0134541.s003.tif]
